# Capturing expert uncertainty: ICC-informed soft labelling for volcano-seismicity

**DOI:** 10.1007/s00445-025-01875-4

**Published:** 2025-09-16

**Authors:** Sam Mitchinson, Jessica H. Johnson, Ben Milner, Oliver Lamb, Yannik Behr

**Affiliations:** 1 School of Environmental Sciences, University of East Anglia, Norwich, UK; 2 School of Computing Sciences, University of East Anglia, Norwich, UK; 3 Earth Sciences New Zealand, Wairakei Research Center, Taupō, New Zealand

**Keywords:** Volcano-seismicity, Inter-rater reliability, Intraclass correlation coefficient, Uncertainty, Ruapehu

## Abstract

Reliable classification of volcano-seismic signals underpins monitoring and eruption forecasting and is an essential tool for advancing understanding of subsurface processes. However, traditional approaches may overlook the inherent uncertainty and variability between expert judgments. We introduce an innovative method that explicitly quantifies inter-expert agreement using the intraclass correlation coefficient (ICC) and incorporates this measure into probabilistic, ICC-informed soft labels, which can be fed into machine learning pipelines. We conducted a global survey involving 89 experts who classified a set of 80 volcano-seismic events from Ruapehu, New Zealand, providing continuous ratings for standard categories: volcano tectonic (VT), hybrid (HYB), long-period (LP), and other (OT). ICC agreement scores revealed that single-rater scores produce poor agreement between experts even for well-established VT and LP classifications. However, reliability significantly improved for these classifications when multiple expert ratings were combined, although, for HYB and OT categories, expert disagreement remained substantial. We developed a soft labelling methodology that weights class probabilities by their respective ICC scores, resulting in a distribution that naturally reflects expert uncertainty. This demonstrates that ICC-informed soft labels could provide a robust alternative to the hard label standard by explicitly capturing classification uncertainty and variability. Our fully probabilistic view has the potential to significantly enhance machine learning model accuracy, robustness, and transferability across volcanic systems and should provide a fundamental shift in how volcano-seismic data are labelled and interpreted within automated monitoring frameworks.

**Supplementary Information:**

The online version contains supplementary material available at 10.1007/s00445-025-01875-4.

## Introduction

Seismology and the classification of volcano-seismic signals are key tools for the monitoring of volcanoes (Ramis et al. 2018). Supervised machine learning (SML) is an effective tool for classification and prediction tasks and is well suited to fields that rely on expert judgement, such as diagnostic healthcare (Garg and Mago 2021; Chhabra and Sharma 2022; Stutz et al. 2023). Naturally, there is an opportunity to use similar techniques within volcanology, where expert judgement is fundamental to the monitoring of volcanoes (e.g. Carniel and Raquel Guzmán 2021). In fact, machine learning techniques adopted from other industries have already shown promise for volcano-seismic monitoring (e.g. Dempsey et al. 2020; Lapins et al. 2021; Manley et al. 2022). Human insight is regularly used for simple manual tasks such as data cleansing for machine learning models (Muller et al. 2021). SML models *learn* through structured labelled data inputs that are split into training and test data. The model then generates predictions on the test data and is calibrated before being applied to unseen data. Consequently, the performance of these models is constrained by the nature of the input data, so the quality of the training data plays a crucial role in machine learning. However, evaluation methods that measure the quality of the data used to train machine learning models are often less sophisticated than the models trained by them (DiPietro and Hazari 2022), and explicit uncertainty quantification for labelled data is generally overlooked (Plank 2022). If the inherent uncertainty in the input data (labels) is not well understood, the output of the model will be less reliable (Northcutt et al. 2021). In contrast, models trained using label uncertainty have been found to improve model performance (Hagenah et al. 2019; Vega et al. 2021; Collins et al. 2022; Tayyab et al. 2023). In this study, through the use of statistical methods developed and utilised in fields dependent on expert judgement, we quantify the variability in volcano-seismic event classification among experts in the field of geophysics and evaluate how this uncertainty can be incorporated into machine learning workflows.

### Volcano-seismic classification

Volcano-seismicity refers to the phenomenon of earthquakes that occur in close proximity to a volcano, typically within 15 km of the active crater, and at shallow depths (i.e. up to 20 km) (McNutt and Roman 2015). These restrictions may be arbitrarily selected to reduce the likelihood of detecting earthquakes from non-volcanic sources and will differ depending on the volcano (Latter 1981). In volcano-seismology, the processing of seismic events is generally divided into the detection and descriptions of the seismic signal characteristics, which can be categorised into discrete groups. Changes in volcanic unrest are often preceded by some kind of volcano-seismic signal (McNutt and Roman 2015), and the characteristics of these signals can be indicative of different volcanic processes (Chouet and Matoza 2013), making the accurate classification of volcanic earthquakes of great importance.

Classifying a volcano-seismic signal is not a straightforward task and is usually performed by local expert analysts (Malfante et al. 2018), sometimes with the assistance of speciality software such as SWARM (Norgaard et al. 2021) or SeisComP (Helmholtz-Centre Potsdam-GFZ German Research Centre For Geosciences and GEMPA GmbH 2008), which are designed to detect, filter, and transform a seismic trace into the frequency and time domain. At volcano observatories, analysts are trained to classify volcano-seismic earthquakes based on characteristics of a trace waveform, spectrogram, and spectrum data. Volcano-seismic classification groups are generally composed of transient and continuous events, each with associated interpretations of the source mechanisms driving the signal (Lahr et al. 1994). Transient events are broadly referred to as high frequency/volcano tectonic (VT) (Latter 1981; Lahr et al. 1994; McNutt 2005), hybrid (HYB) (Lahr et al. 1994; Chouet and Matoza 2013), and low frequency/long-period (LP) (Aki and Koyanagi 1981; Lahr et al. 1994). Modern higher-precision technology has led to the discovery of very-long-period (VLP) (Neuberg et al. 2000; Zoback et al. 2013) and even ultra-long-period (ULP) events (Coppess et al. 2022), which have dominant energy for frequencies <0.1 Hz. Volcanic tremor is a description of a continuous earthquake signal; however, the terminology can be used to describe a variety of seismic signatures in geophysics, which can be confusing. Other indirect volcano-seismic signals, such as explosions and rockfalls, are also commonly recorded in proximity to volcanoes. This study will focus on the classification of transient earthquake signals as described.

The implications that volcano-seismic source models have for a volcanic system are of great importance for volcano monitoring. A VT event is indicative of a brittle failure response indirectly linked to processes in the volcanic system, such as a magma intrusion (Roman and Cashman 2006). LP events are most commonly associated with fluid movement within a crack or conduit (Chouet 1996). HYB earthquakes have characteristics of both VT and LP events, typically described as a VT onset with a coda similar to LP, which is often interpreted as a manifestation of an interaction between brittle failure intersecting with a fluid-filled crack or conduit (Lahr et al. 1994). However, the term has also been used to include LP-like events with any appreciable high-frequency energy (Neuberg et al. 2000), and a range of alternative source mechanisms have been hypothesised (e.g. Lahr et al. 1994; Neuberg et al. 2000; Harrington and Brodsky 2007). Given this variability in both waveform characteristics and physical interpretations, it is likely that HYB events will be more difficult for analysts to classify consistently.

Machine learning techniques for automating the classification of volcano-seismic signals have been studied extensively (e.g. Scarpetta et al. 2005; Langer et al. 2006; Curilem et al. 2017; Malfante et al. 2018; Manley et al. 2022; Ferreira et al. 2023; Zhang et al. 2024). Despite promising results, these methods have yet to be standardised or integrated into volcano monitoring practices. Previous models have been trained using labelled data prepared by a single expert in a controlled research setting (e.g. Curilem et al. 2009). These labels are constructed without consideration of the agreement between experts, sometimes referred to as *inter-rater agreement* (Fleiss 1971), which is problematic because the classification of volcano-seismic events can vary considerably depending on the opinion of the expert (Chouet and Matoza 2013; Duque et al. 2020; Vyas et al. 2021). Agreement statistics have been utilised in volcano-seismic classification to evaluate the outputs of the model (Canário et al. 2020). However, it is difficult to evaluate the reliability of models when there is unknown systematic uncertainty contained in the model inputs. Indeed, training data labelled by a single expert could considerably bias the model towards the views of the expert (Stutz et al. 2023; Le et al. 2023), preventing the model from being able to generalise well to other settings (Vyas et al. 2021). Furthermore, it has been found that the most common root of model error is due to the result of label error (Linville et al. 2019), highlighting the importance for capturing uncertainty in SML training data.

### Ground truth—data labelling

SML models interpret training data labels as correct or *ground truth* outcomes that the model uses to learn from the data, emphasising the importance of accurate training data labels (Muller et al. 2021). Training SML models requires a large volume of labelled data. Outsourcing the labelling process by contracting external teams or platforms, such as Amazon Mechanical Turk (MTurk) (e.g. Mortensen and Hughes 2018; Aguinis et al. 2021), is becoming an increasingly popular strategy for managing data annotation tasks (Aguinis et al. 2021). Outsourcing can serve as an efficient approach when it comes to large-scale processing of straightforward data set labelling (Ahfock and McLachlan 2021), but it is inadequate for labelling tasks that require expert knowledge. The primary obstacles to annotating data sets dependent on expert knowledge are time and expense (Elmes et al. 2021; Le et al. 2023), and consequently, training SML models with quality expert-level data presents a significant challenge within the field of machine learning.

Human error and uncertainty are natural and unavoidable occurrences. In expert judgement, the level of disagreement (variability) between raters is called noise (Kahneman et al. 2021). Ground truth is often assumed in machine learning training data, and the quantification of noise in human label variation is overlooked (Plank 2022), which inevitably leads to an unknown overestimation of the model’s capabilities to make predictions (Frenay and Verleysen 2014; Schmarje et al. 2022; Stutz et al. 2023). Indeed, machine learning studies across various disciplines often fail to report on the quality or the methods for human-labelled training data altogether (Geiger et al. 2021). There are methods exploring the use of unsupervised machine learning to help with the data labelling process in volcano-seismology (e.g. Cui et al. 2021, 2024), but these still require the intervention of an expert. To obtain high-quality labelling for data that require expert evaluation, it may be a good strategy to quantify the level of agreement between experts, as a form of uncertainty (Hagenah et al. 2019; Jiang and Nachum 2019; Tayyab et al. 2023). This could be achieved by requiring experts to provide probability distributions for the class labels, a method commonly known as *soft labelling* (Quost et al. 2017; Silva and Oliveira 2021; Vega et al. 2021; Collins et al. 2022; Grossmann et al. 2022; Nousi and Tefas 2024). Machine learning and deep learning models are capable of incorporating uncertainty into model predictions through soft labelling and other methods (e.g. Tayyab et al. 2023; de Vries and Thierens 2024). Recent advances in uncertainty-aware ML in the geosciences have shown similar benefits when uncertainty is encoded through label smoothing (e.g. Alfaro-Diaz et al. 2025) or Bayesian ensemble methods (e.g. Myren et al. 2025) in seismic event classification, both of which are beyond the scope of this study. Label benchmarking studies have shown that classifiers trained on soft labels repeatedly outperform models trained on discrete hard labels, particularly for smaller and imbalanced data sets (Madani Tonekaboni et al. 2020; Grossmann et al. 2022; Schmarje et al. 2022; de Vries and Thierens 2024). Furthermore, eliciting soft labels for learning has been shown to improve model performance while relying only on a few annotators (Collins et al. 2022). Yun et al. (2021) showed that transforming the training data from a single label to soft label improved accuracy from +1.4 percentage points (pp) to +2.6 pp, and robustness up to +8.7 pp. Peterson et al. (2019) calculated frequency vectors over crowdsourced human annotators to create soft label targets from the CIFAR-10 image data set (e.g. Krizhevsky 2009). The study found that the soft labelled CIFAR-10 H training data improved accuracy for CIFAR-10 and ImageNet-Far by +1.0 pp and +2.0 pp, respectively, while reducing the cross-entropy considerably.

**Fig. 1:**
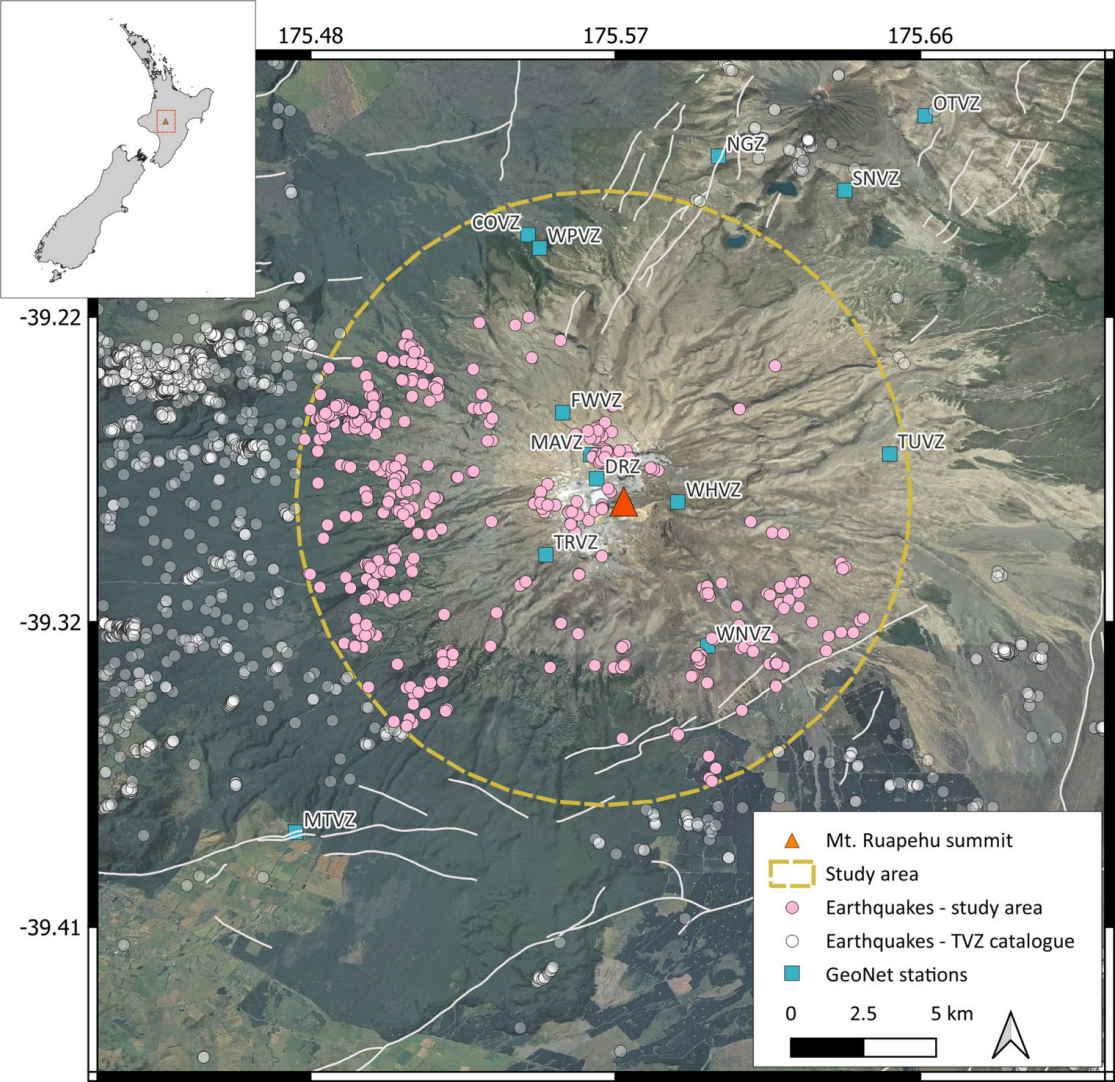
A map of the Ruapehu volcano region study area, located in the New Zealand North Island as shown by the inlay. The Ruapehu study area shows active fault lines (Langridge et al. 2016) with relocated earthquakes from the TVZ catalogue and stations that were active from 2007 to 2024 (DRZ and WPVZ are no longer active). The study area was selected to contain earthquake events attributed to Ruapehu and contains 473 shallow events (M$_c$=0.3). New Zealand Imagery was accessed on 12/06/2025 from https://registry.opendata.aws/nz-imagery. Licensed by AWS for re-use under CC-BY$-$4.0

We believe that statistical methods can be used to combine the views of experts and create a soft labelled data set that harnesses the inevitable uncertainty in expert judgement. For volcano-seismic classification, the level of agreement between expert judgement is quantified for the first time, and a method for constructing soft labels from annotator uncertainty is proposed. The downstream impacts on machine learning model reliability, robustness, and generalisation are beyond the scope of this study, but are likely to be beneficial (Peterson et al. 2019; Yun et al. 2021; Collins et al. 2022; de Vries and Thierens 2024). The specific objectives for this study are (1) to quantify the agreement between experts on volcano-seismic signatures using the intraclass correlation coefficient (ICC) (Shrout and Fleiss 1979) and (2) to develop an agreement-informed soft labelling method suitable for volcano-seismic classification. The study collated data by crowdsourcing expert judgement globally through the use of an online questionnaire, harnessing inter-assessor agreement statistics to develop soft labels for volcano-seismic signatures.

## Methods

The method for this study involves the construction of a survey aimed at gathering the judgements of experts in the field of geological science through an online questionnaire. The questions were designed to obtain a classification for volcano-seismic signals based on predefined classification criteria that align with the standards of most volcano-seismologists. This section is divided into three parts: First, we provide an overview of the research area and data collection. Following that, we detail the design and administration of the questionnaire. Finally, we define and explain the statistical techniques used to interpret the results.

### Data

The earthquake catalogue used for this study is a subset of a larger catalogue of relocated events in the Taupo Volcanic Zone (TVZ), New Zealand, from 2007 to 2024 (Illsley-Kemp and Mestel 2025). The area of interest is a 9.5 km radius circle with the summit of Ruapehu volcano as the centroid, designed to encapsulate the volcano and exclude events associated with regional tectonic activity and neighbouring volcanoes (Fig. 1). Ruapehu is a popular tourism spot with hundreds of tourists visiting the Tongariro National Park daily and thousands of people skiing on the flanks of the volcano. The eruptive behaviour at Ruapehu is characterised by periodic low-volume (< 0.05 km$^3$), but sudden phreatomagmatic eruptions that occur frequently (every 25–30 years) (Kilgour et al. 2013; Conway et al. 2016), with major magmatic eruptions occurring every 50 years on average, the most recent being the 1995/1996 sequence (Bryan and Sherburn 1999; Hurst and McGinty 1999; Sherburn et al. 1999).

The Illsley-Kemp and Mestel (2025) relocated earthquake catalogue was constructed using the EQTransformer model (Mousavi et al. 2020), which was trained on earthquake datasets, which exclude low-frequency events, meaning the resulting catalogue may bias the events towards the higher-frequency events. We preferred the catalogue by Illsley-Kemp and Mestel (2025) over the GeoNet catalogue because it contains more low-magnitude events in the proximity of the volcano summit. The earthquake catalogue was filtered to only contain shallow earthquakes (i.e. <20 km) because we were interested in earthquakes most likely to be associated with volcanic processes. The magnitude of completeness (M$_c$) was calculated by fitting the Gutenberg-Richter relationship to the cumulative magnitude count and estimating the point of maximum curvature (Wiemer 2000).

The vertical component raw seismic data streams were downloaded from the GeoNet FDSN client (GNS Science 2021) using the onset time recorded in the TVZ relocated catalogue (Illsley-Kemp and Mestel 2025). To minimise the effects of seismic attenuation, the closest station to the event epicentre was selected, within a maximum radius of 10 km. A 20 s time window was selected for the trace signal, allowing a buffer for inaccurate P-wave onset calculations by starting 0.5 s before the picked onset time. The trace was filtered using a Butterworth bandpass at 1–25 Hz and resampled to 50 samples per second. Finally, a Hanning taper was applied to remove abrupt edge discontinuity when slicing the trace signal by multiplying the trace slice by a cosine-shaped taper, which suppressed spectral leakage caused by microbarom or microseismic noise (e.g. Behr et al. 2013; De Carlo et al. 2021), whilst preserving the earthquake signal. For the spectrogram, we defined a 1.5 s window length with an overlap of 85% to improve the time resolution and computed the short-time Fourier transform (STFT). The amplitude spectrum was created by transforming the time-domain signal into the frequency domain using the fast Fourier transform (FFT), then isolating and normalising the positive-frequency components. These parameters produced clear static plots that support interpretation and closely resemble data streams in specialist software, such as SWARM (Norgaard et al. 2021).

### Development of the questionnaire

Online surveys provide an effective approach for crowdsourced data, particularly as experts reside in many different regions around the world. We collaborated with subject-matter experts at Earth Sciences New Zealand (formerly GNS Science) to develop an online questionnaire that could be sent by email to other experts around the world. An important component of the questionnaire was to use a structured input format to collect the opinions of a variety of experts to classify a volcano-seismic event into predefined categories, based on the visual and descriptive data consistent with their day-to-day work. Given that the purpose of the study was to assess the agreement between experts on current classification regimes, we limited the criteria to the following labels: VT, HYB, and LP, as defined by McNutt and Roman (2015). We omitted *tremor* from the standard classifications, in part due to disagreement on terminology and partly because we decided to focus on transient earthquake signals in this study. We did not include very low-frequency signals (i.e. <0.1 Hz) because these fall below the bandpass filter (1 Hz). We assumed that the earthquake events in the catalogue are geophysical phenomena and not of anthropogenic origin, and we did not include a standard label for explosions or rockfalls, as these can be relatively rare events. However, participants did have the option of labelling the signal as *other* (OT) and writing a description. To keep the layout of the questionnaire clear and easy to interpret, we avoided using multiple stations and channels, which would overcrowd the webpage. However, we understand that some experts may use data from multiple stations to help classify an event. Instead, we selected a single channel from stations located near the volcano summit and produced waveform, spectrogram, and amplitude spectra graphs. We decided to anonymise where volcano-seismic events originated and to limit the contextual information on the event to magnitude, depth, and the proximity of the epicentre to the nearest station. This was done to reduce an inherent bias that may be present in experts who had previously worked in the region.

**Fig. 2:**
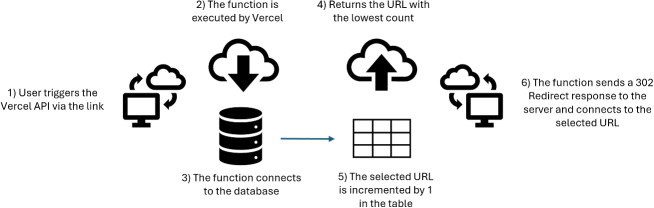
A computer programming methodology for distributing the batches of questionnaires evenly. The API hosted by Vercel was triggered when a participant clicks the link, which looks up the batch URL with the fewest visits and returns that URL for the user

When classifying each event, we decided to use a continuous scale to minimise the risk of selection bias that could arise from using a nominal or ordinal scale. This approach allows one to derive a probability distribution from the participants’ responses. The likelihood scores for each volcano-seismic classification were collected using sliders, allowing participants to provide more refined answers. The likelihood scoring is calculated with a bipolar Likert scale, where a score of $+1$ suggests the expert is certain that the event fits the category, a score of $-1$ suggests certainty that the event does not fit the category, and a score of 0 would be selected if the expert is impartial. Using bipolar Likert scale data to create a distribution provides novel insights into consensus and variability in volcano-seismic classification assessments. The questionnaire included a question asking participants to rate the usefulness of the data streams when classifying the signals on a scale of 1 to 10. It also collected information on the professional backgrounds of the participants, such as their job title, years of experience, and the volcanic regions they have worked in, which were used to make further comparisons on the agreements between experts. Full illustrative examples of the questionnaire are available in the supplementary material.

Although there are methods to calculate the appropriate sample size for agreement statistics (e.g. Koo and Li 2016), it was difficult to forecast the total number of completions or control any response bias. However, we did consider the chances of participant fatigue or loss of interest in longer surveys, leading to unreliable answers or even incompletion (Sharma 2022). Response and completion rates can vary considerably depending on the length, content, and mode of administration (Booker et al. 2021; Sharma 2022). In survey research, completion rates are higher for shorter surveys than longer ones, and the data quality of participant responses is also greater for shorter surveys (Sahlqvist et al. 2011; Kost and Rosa 2018). Surveys containing 13 questions have been found to have a completion rate of 63%, with completion rates dropping to 51% for surveys containing 25 questions (Kost and Rosa 2018). Trial questionnaires were sent to a small pool of participants to receive feedback on the length of the questionnaire, and a general consensus of 10 classification questions with three additional introductory questions asking for participant background was deemed long enough to achieve a statistically significant pool of responses, whilst maximising the chance of participant completion.

We created eight questionnaires, each containing 10 unique earthquakes (80 unique events in total). Our aim was to ensure that each expert group classified a similarly diverse set of volcano-seismic signals, without overlap between questionnaires. As ground truth labels were unknown *a priori*, we grouped events using the frequency index (FI) (Buurman and West 2010) into low, mid, and high-frequency strata, and a fixed quota was drawn from each:

1 $$\begin{aligned} \text {FI} = \log _{10} \left( \frac{\bar{A}_{\text {upper}}}{\bar{A}_{\text {lower}}} \right) , \end{aligned}$$

where the $\bar{A}_\text {upper}$ and $\bar{A}_\text {lower}$ are the mean spectral amplitudes in the predefined bands. LP earthquakes in volcanic regions have been shown to contain the majority of seismic energy for frequencies $<=$5 Hz (Lahr et al. 1994; Chouet 1996; Neuberg et al. 2000). Therefore, for the FI calculation, we used a high-frequency band of 5–20 Hz and a low-frequency band of 1–5 Hz. The sampled events were representative of the FI distribution in the catalogue and guaranteed exposure to both high and low-frequency signals within a 10 question batch (see supplementary material). Illsley-Kemp et al. (2022) found for earthquakes within the TVZ, FI decreases with distance from the earthquake source due to path effects. We found that using the closest station to the source produced a similar FI distribution to the attenuation-adjusted FI in Illsley-Kemp et al. (2022), so we therefore decided no additional corrections were necessary.

We targeted four specialist volcanology and geophysics email chains containing >1000 international and domestic (UK) professionals and early career researchers. After following the link, each participant would be assigned one of eight batches containing 10 earthquake events. Unknown biases, such as imbalanced ratios of early-stage researchers to senior researchers and individuals with more time availability, could not be controlled. However, we developed a programming methodology via an API call to evenly distribute the questionnaire batches sequentially, determined by when the user clicks on the link (Fig. 2). This also means that we were less likely to bias responses based on time zones.

### Inter-rater reliability

The pooling of expert judgement is an established problem in volcanology (e.g. Clemen and Winkler 1999; Aspinall et al. 2003; Aspinall 2010). When analysing the classifications scored by multiple experts, it would be ideal for them to score similarly to each other so that we can be confident that their scores reflect the *true* label. In practice, variability due to noise is inevitable, and a single-rater design conceals this variability (Kahneman et al. 2021). Variability can be revealed by the construction of a noise audit consisting of multiple annotators, which can be statistically tested using the intraclass correlation coefficient (ICC) (Shrout and Fleiss 1979). ICCs have been favoured to assess absolute agreement between raters, particularly in the medical sciences (Spence Laschinger 1992; Wu et al. 2012; Carlsson et al. 2017), where noise between expert judgements can be high (Chen et al. 2017; Hagenah et al. 2019). There are six original ICC forms for reliability studies (Shrout and Fleiss 1979), in which the classes are agreed upon by experts (raters) and are distinguished based on their values for A and B in ICC(A, B). Variable A denotes the model, which can be a one-way random (model 1), two-way random (model 2), or a two-way mixed model (model 3). For model 1, each volcano-seismic event would be rated by a different set of raters, model 2 is a random sample of judges providing ratings for *n* events, and for model 3, the same *k* raters are used for *n* events (Shrout and Fleiss 1979). Variable B describes whether the scores are averaged or not, where *k*, which is a function of B, is the total number of raters in the set. For this study, experts are grouped into different batches and rate a unique set of volcano-seismic events, and these experts are randomly sampled from a global pool of experts. ICC(2,k) calculates absolute agreement on the performance of multiple annotators in the belief that the background expertise of the participants is relevant to the task being performed and also assumes that the raters are randomly selected from a larger pool of experts (Trevethan 2017). Therefore, we propose using ICC(2,k) to assess the overall expert agreement across each batch of events and the ICC(2,1) method to judge whether a single-rater score is reliable. The ICC(2,k) calculation is outlined by Shrout and Fleiss (1979) as

2 $$\begin{aligned} \text {ICC}(2,k) = \frac{MSR - MSE}{MSR + \frac{MSC - MSE}{n}} \end{aligned}$$

where *k* is the number of raters for each batch, *MSR* is the mean square of the scores for all events in the batch, *MSC* is the mean square for raters’ scores, *MSE* is the mean square error (residual), and *n* is the number of events (10). Here, we show that ICC(2,k) is a two-way random-effects model calculating absolute agreement by the mean of *k* raters (Shrout and Fleiss 1979). A detailed guide on how the ICC(2,k) parameters were calculated is available in the supplementary material. One can also use the ICC(2,1) to show the reliability of a single rater within a given set of raters to assess whether we could reliably, on average, use a single rater to perform volcano-seismic classification:

3 $$\begin{aligned} \text {ICC}(2,1) = \frac{MSR - MSE}{MSR + (k - 1) MSE + \frac{k(MSC - MSE)}{n}} \end{aligned}$$

ICC agreement scores are generally bound between [0, 1], with 1 symbolising perfect agreement and a score of 0 indicating that agreement is no better than random chance. We adhere to the *rule of thumb* (e.g. Koo and Li 2016) where ICC values <0.5 are indicative of poor reliability, values between 0.5 and 0.75 indicate moderate reliability, values between 0.75 and 0.9 indicate good reliability, and values >0.90 indicate excellent reliability.

### Construction of training data

Within the questionnaire, we designed a continuous bipolar Likert scale [$-1$, 1] with 0 reflecting neutrality. Expert scores extracted from the questionnaire were stored as a table with columns *Batch, Event, Rater, VT, LP, HYB, OT*. We used a logistic-normal MAP framework (maximum *a posteriori*) to construct soft labels from the continuous expert scores $s_{e,r,c}\in [-1,1]$ representing rater $r$ and class $c$ for each event $e$. This framework builds upon concepts in Bayesian label aggregation (e.g. Dawid and Skene 1979; Clemen and Winkler 1999). Our framework is modelled as a Gaussian-distributed observation of a latent logit $\phi _{e,c}$:

4 $$\begin{aligned} s_{e,r,c} \;\sim \; \mathcal {N}\!\bigl (\phi _{e,c},\,\tau ^2\bigr ). \quad \tau >0, \end{aligned}$$

The latent variable (logit) is indirectly derived from the inherently noisy annotations ($s_{e,r,c}$) and represents the strength of the relationship between instance and class. Logit $\phi _{e,c}$ is the *true* log-preference for class $c$ in event $e$, and $\tau$ captures the overall rater variability. With a flat prior on $\phi$, the posterior mode (MAP) (Eq. 4) is simply the sample mean of the scores (Bishop 2006). We then scale by the mean inter-rater reliability (ICC score) ($\rho _{b,c}$) of the batch $b$ for each class $c$:

5 $$\begin{aligned} \hat{\phi }_{e,c} = \rho _{b,c}\;\frac{1}{k}\sum _{r=1}^{k} s_{e,r,c}, \qquad \rho _{b,c} = \max \!\bigl (0,{\text {ICC}}(2,k)_{b,c}\bigr )\in [0,1], \end{aligned}$$

where ICC(2,k)$_{b,c}$ is the intraclass coefficient for $k$ raters. Clamping negative ICCs to zero ensures that classes with poor agreement contribute nothing to the final label. Finally, by combining all per-class logits for event $e$ into a single vector $\hat{\phi }_e=(\hat{\phi }_{e,1},\dots ,\hat{\phi }_{e,C})$, the class probabilities are constructed using a temperature-controlled (*T*) *softmax* function (Hinton et al. 2015):

6 $$\begin{aligned} p_{e,c} = \frac{\exp \!\bigl (\hat{\phi }_{e,c}/T\bigr )}{\displaystyle \sum _{j=1}^C\exp \!\bigl (\hat{\phi }_{e,j}/T\bigr )}, \quad T>0. \end{aligned}$$

Dividing by $T$ adjusts the confidence of the distribution, where $T<1$ enhances the sharpness of the distribution, and $T>1$ increases the uniformity of the distribution. This gives the resulting $\sum _c p_{e,c}=1$ and $p_{e,c}\in (0,1)$ for our final soft labels. We found that the default $T=1$ yielded overly uniform labels compared to the raw scores. Through testing different temperature scales in the range [0.1, 1], we found that a temperature scaling of $T=0.3$ produced soft labels that captured the underlying spread of the data.

**Table 1:** Distribution of participants by years of experience across batches

Batch name	Experts	Student	$<1$ year	1–5 years	5–10 years	10–20 years	$>20$ years
b1	12	–	–	2	3	3	4
b2	10	–	–	1	2	7	–
b3	10	2	–	2	1	2	3
b4	10	–	–	2	2	4	2
b5	11	–	–	2	2	4	3
b6	12	–	1	2	4	3	2
b7	13	1	1	1	2	3	5
b8	11	–	1	2	3	2	3
Proportion		3%	3%	16%	22%	31%	25%

The step-by-step process is detailed in algorithm 1. Each batch $b$ contains $n$=10 events with four classes $c$. The same $k$ experts score every event-class within their batch. Class-specific reliability weights are calculated on the full matrix as $\rho _{b,c}=\max \!\bigl (0,{\text {ICC}}(2,k)_{b,c}\bigr )$. Finally, we scale the per-class sample means by $\rho _{b,c}$ to obtain logits, then apply a temperature-scaled soft-max ($T = 0.3$) to produce a four-class probability vector for each event that sums to one.

**Algorithm 1:**
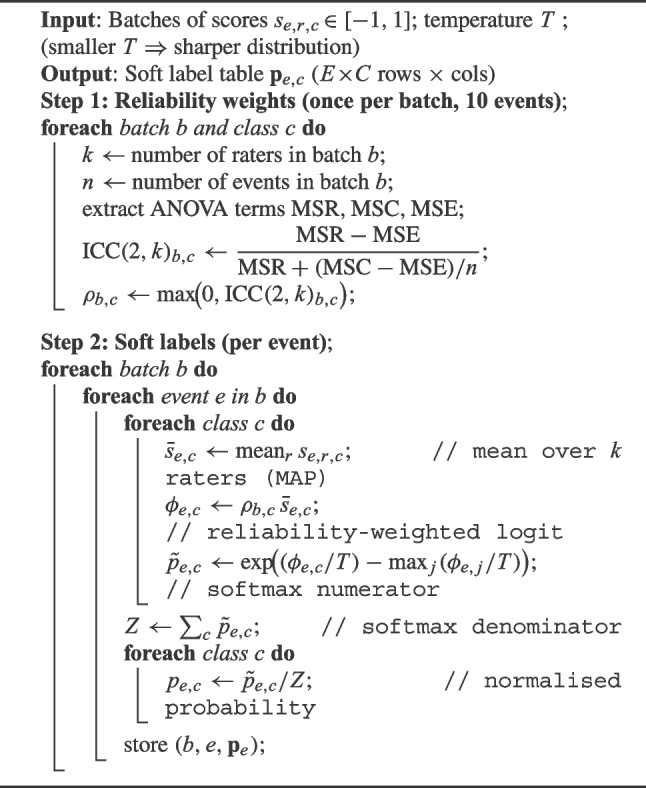
Soft label construction from continuous bipolar ratings.

## Results

The methodology for obtaining response rates was based on website hits via the API call, which may be inaccurate if a user revisits the page or clicks the link multiple times. However, we estimate that the online questionnaires received a total of 488 visits over a period from March to May 2025, with a total number of 89 submissions across all eight batches (890 classifications), giving an approximate completion rate of 18%. We downloaded each batch from the online form repository and transformed the data into a single repository. The questionnaire yielded an even spread of completions for each batch, with a range of experience from postgraduate students to >20 years of experience. Table 1 shows a uniform distribution of participants for each batch from early career to senior experts, with 31% of experts within the 10–20-year category. Batch 2 shows the least diversity in experience, with 70% of the participants having 10–20 years of experience. In general, the pool of experts had a diverse range of professional backgrounds but with a focus on volcanology and seismology, 32% identified as volcano-seismologists, 29% seismologists, and 23% volcanologists. A total of 82% of participants had previous experience classifying volcano-seismic events as part of their work. The vast majority of the participants had worked at a volcano for at least 1 year, and many experts noted experience in multiple volcanic regions. The spread of expert experience per region was relatively uniform, with the most frequently worked region (17% of participants) being the Southwest Pacific and the least (5% of participants) being Antarctica. In summary, this distribution of experience suggests that the data set captures insights from scientists at varying career stages, encompassing both early-career and seasoned professionals, while also reflecting a wide range of geographical and specialist backgrounds. In this section, we quantify the level of agreement between participants within each batch for class labels and construct a probabilistic soft labelled data set.

**Table 2:** Expert agreement: single-rater ICC(2,1) and average-rater ICC(2,*k*) with 95% CIs for *k* number of experts

	ICC(2,1)				*k*	ICC(2,*k*) [95% CI]			
Batch	VT	HYB	LP	OT		VT [95% CI]	HYB [95% CI]	LP [95% CI]	OT [95% CI]
b1	0.54	0.12	0.42	$-$0.02	12	0.94 [0.72-$-$0.96]	0.63 [$-$0.17-$-$0.77]	0.90 [0.60-$-$0.94]	$-$0.27 [$-$0.57-$-$0.01]
b2	0.58	0.12	0.47	0.00	10	0.93 [0.74-$-$0.96]	0.59 [$-$0.07-$-$0.78]	0.90 [0.72-$-$0.95]	$-$0.01 [$-$0.03-$-$0.00]
b3	0.41	0.17	0.41	0.10	10	0.87 [0.71-$-$0.91]	0.68 [$-$0.45-$-$0.85]	0.88 [0.48-$-$0.92]	0.53 [$-$0.06-$-$0.61]
b4	0.58	0.11	0.63	0.10	10	0.93 [0.75-$-$0.97]	0.55 [$-$0.11-$-$0.72]	0.94 [0.36-$-$0.98]	0.52 [$-$0.09-$-$0.69]
b5	0.46	0.09	0.52	0.06	11	0.89 [0.60-$-$0.94]	0.51 [$-$0.14-$-$0.70]	0.92 [0.35-$-$0.95]	0.41 [0.01-$-$0.64]
b6	0.44	0.02	0.32	0.03	12	0.90 [0.76-$-$0.94]	0.19 [$-$0.68-$-$0.57]	0.85 [0.64-$-$0.90]	0.27 [$-$0.00-$-$0.42]
b7	0.43	0.13	0.40	$-$0.00	13	0.91 [0.64-$-$0.95]	0.66 [ 0.19– 0.74]	0.90 [0.33-$-$0.94]	$-$0.02 [$-$0.09-$-$0.02]
b8	0.36	0.04	0.29	0.01	11	0.86 [0.69-$-$0.91]	0.31 [$-$0.81-$-$0.62]	0.82 [0.19-$-$0.91]	0.11 [$-$0.05-$-$0.26]

**Fig. 3:**
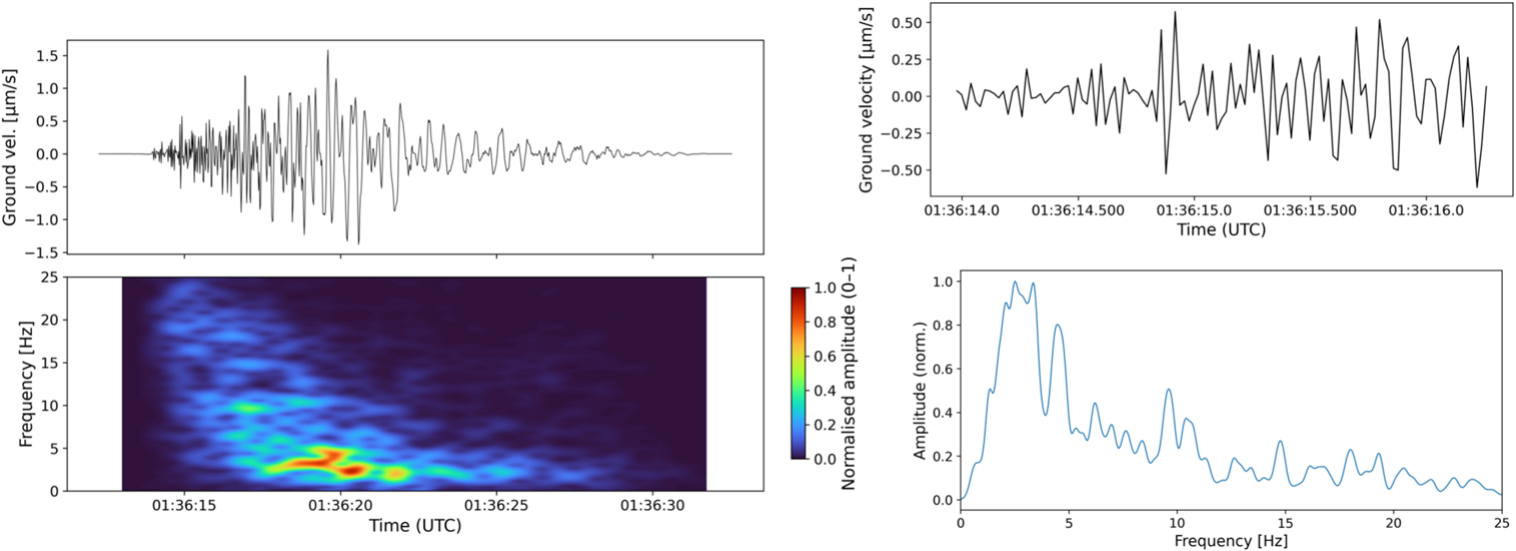
Question 6 from batch 6 shown in trace, onset zoom, spectrogram, and spectrum form. The Mw 0.6 event epicentre was approximately 3.1 km from COVS station (on the northern flank of Ruapehu) at 01:36:12UTC on 07/10/2012 at a depth of 4.2 km

### Expert agreement

We present point estimates and confidence bands for the ICC as an indicator of expert (rater) agreement for the classification of volcano-seismic events at Ruapehu (Table 2). For each batch, the reliability of a single rater yields moderate to low ICC scores. ICC(2,1) is particularly low for HYB and OT classifications, indicating that the scores from the raters fluctuate significantly. Both point estimates and confidence bands approach zero and often include negative values, indicating significant disagreement between raters. Averaging raters improves agreement point estimates for HYB events across batches, but the lower confidence band still includes values <0, meaning there is no stable between-event consensus. ICC point estimates remain poor for most batches for HYB even when averaging the raters, confirming the lack of reliability in the rater scores.

We observe a significant improvement in agreement when estimating absolute agreement between the average ratings, which means that more raters can dampen the noise in imprecise single-rater scores, as expected (e.g. Koo and Li 2016). For a single expert, we could expect VT classifications to be 35–58% reliable; however, we see a significant improvement when averaging across all raters in each batch to 83–94% reliability. A similar improvement is noted for the reliability of LP classifications, increasing from 29–63% to 80–94%. According to the common practice (e.g. Koo and Li 2016), this is classified as a good–excellent agreement reliability and stability for the classification of VT and LP events when averaging across all experts ($k>9$). HYB events generally achieve moderate agreement scores, but there is significant variability between batches; in particular, *b6* returned very poor agreement, even when averaged across 12 experts.

Figure 3, sampled from questionnaire batch 6, illustrates an example of an event that produced noisy scores. In fact, the differences between the scores were considerable for all classes, but especially for HYB. The standard deviation for the raw annotator scores in b6 for HYB classifications is consistently high ($>=$0.44) for all events with a maximum standard deviation of 0.75. This inconsistency in scores for HYB events results in very low per-rater and average ICC scores for HYB. The raw data showed that five experts had absolute certainty (a score of 1) that this was a HYB signal. Two experts believed with absolute certainty that this was not a HYB signal (with a score of $-1$). Two other experts noted that they were neutral for the HYB class (a score of zero), and both believed that it was actually an LP event. The final three experts submitted more conservative scores both for and against the likelihood of the signal being a HYB. This large within-event variance (MSE) is what drives the poor agreement ICC score for b6 and shows that we cannot reliably build interpretations from a single sampled score from this batch for HYB events.

**Fig. 4:**
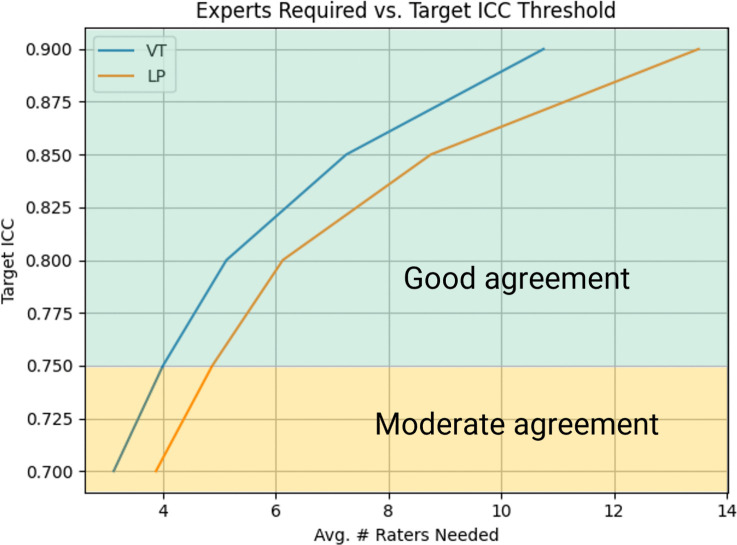
A line graph showing the *k* number of experts and projected agreement score using the Spearman-Brown formula. We observe a more pronounced increase in agreement by increasing the number of experts, up to around 7–8 individuals, at which point the gain in ICC becomes less substantial with each additional rater

In summary, single-rater scores range from poor to moderate reliability for VT and LP labels and are unreliable for HYB and OT. Increasing the number of raters improves VT and LP to good–excellent reliability and improves HYB labels to moderate reliability across most batches. We used the Spearman-Brown formula to sample agreement scores for increasing *k* raters to find the minimum number of raters required to achieve a desired ICC score. We found that at least 4–5 experts on average are required to achieve good agreement for VT and LP classifications, and for both event classes, the projected ICC increases notably with the first 7–8 raters, after which adding more raters results in diminishing returns. This indicates that beyond approximately eight experts, each additional expert contributes incrementally less to the overall inter-rater reliability (Fig. 4). Increasing the number of experts does not improve agreement for HYB and OT labels.

**Fig. 5:**
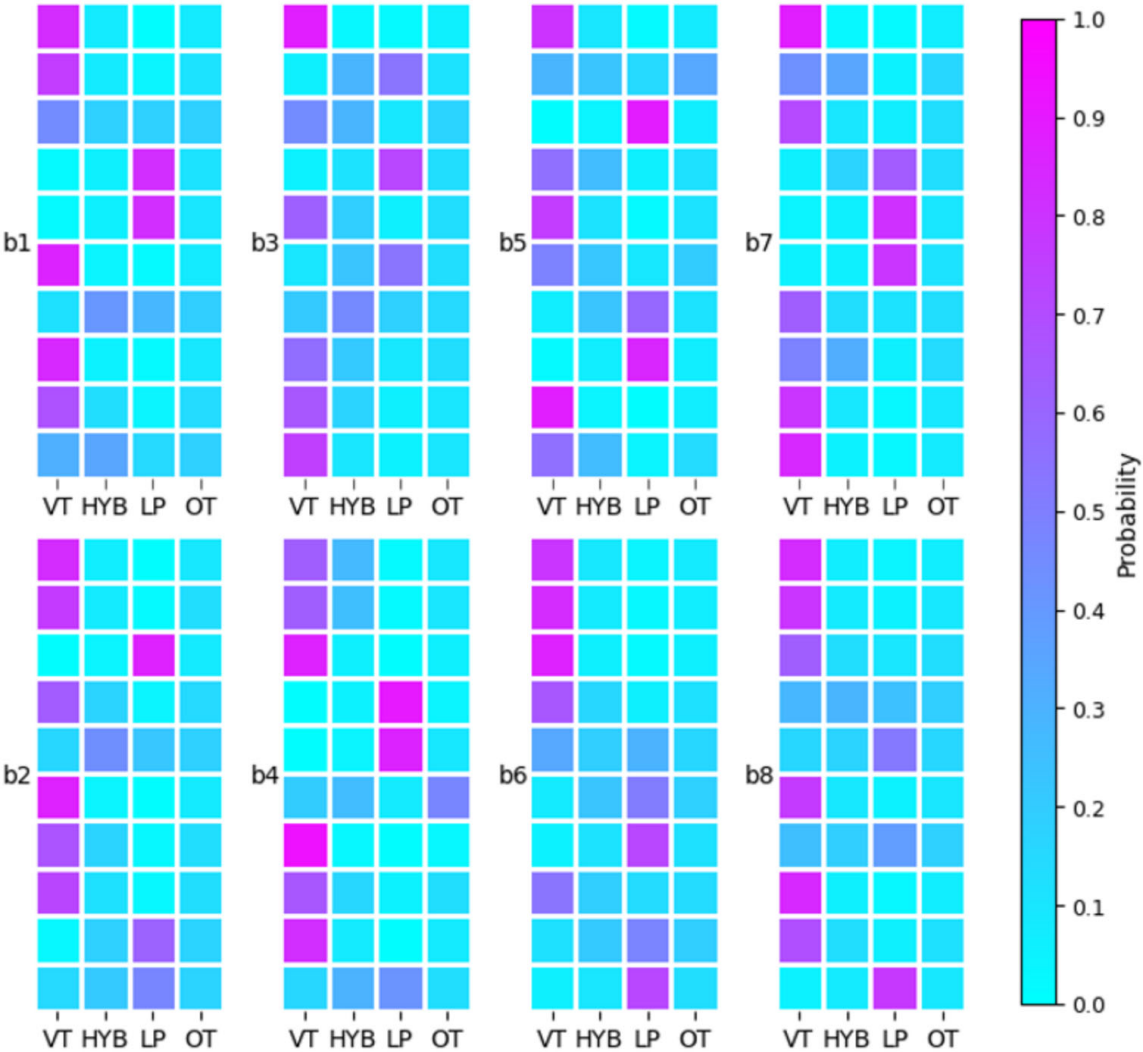
A heatmap showing the complete set of probabilistic soft labels calculated from expert scoring. The catalogue is grouped into eight sub plots and each batch consisting of 10 events. Within sub plots, each row represents an event, with the columns representing the class labels [VT, HYB, LP, OT]. A full table of soft label outputs is available in the supplementary material

**Fig. 6:**
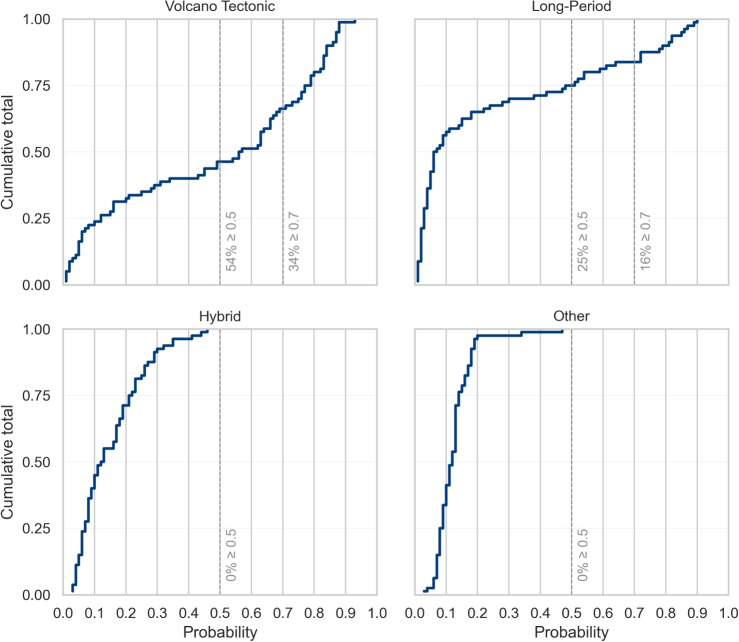
The cumulative total of events and associated soft label probability for each classification across all 80 events

### Soft labels

Figure 5 illustrates the comprehensive soft labelled data set that encompasses all events recorded in the questionnaire. The inclusion of the ICC(2,k) down-weights categories where there is rater disagreement, which results in lower probabilities for HYB and OT classes in general (Fig. 6). In fact, the probability of an event belonging to the HYB or OT classification is never >50%. Whereas 54% of events were believed to have a >50% probability of being a VT, and 34% of events had a >70% probability of being a VT. LP events had a similar distribution to VT, where 25% of events were considered to have a >50% probability of being an LP and 16% of events were considered to have a >70% probability of being an LP (Fig. 6). The distribution of soft labelled VT events follows a bimodal distribution suggesting that the experts were confident that around half of the catalogue was a VT and half of the catalogue was a different kind of event. This is somewhat similar for LP; however, there are far fewer events in the catalogue matching this description. Although agreement was still high, the experts tend to score more conservatively for LPs compared to VTs. The distribution of HYB classifications is right-skewed with the bulk of probabilities 0.1–0.3, due to low-confidence in the ICC scores. The OT category has a tight cluster of low-probabilities with very few outliers, showing that these are comparatively rare events and also not well defined.

**Fig. 7:**
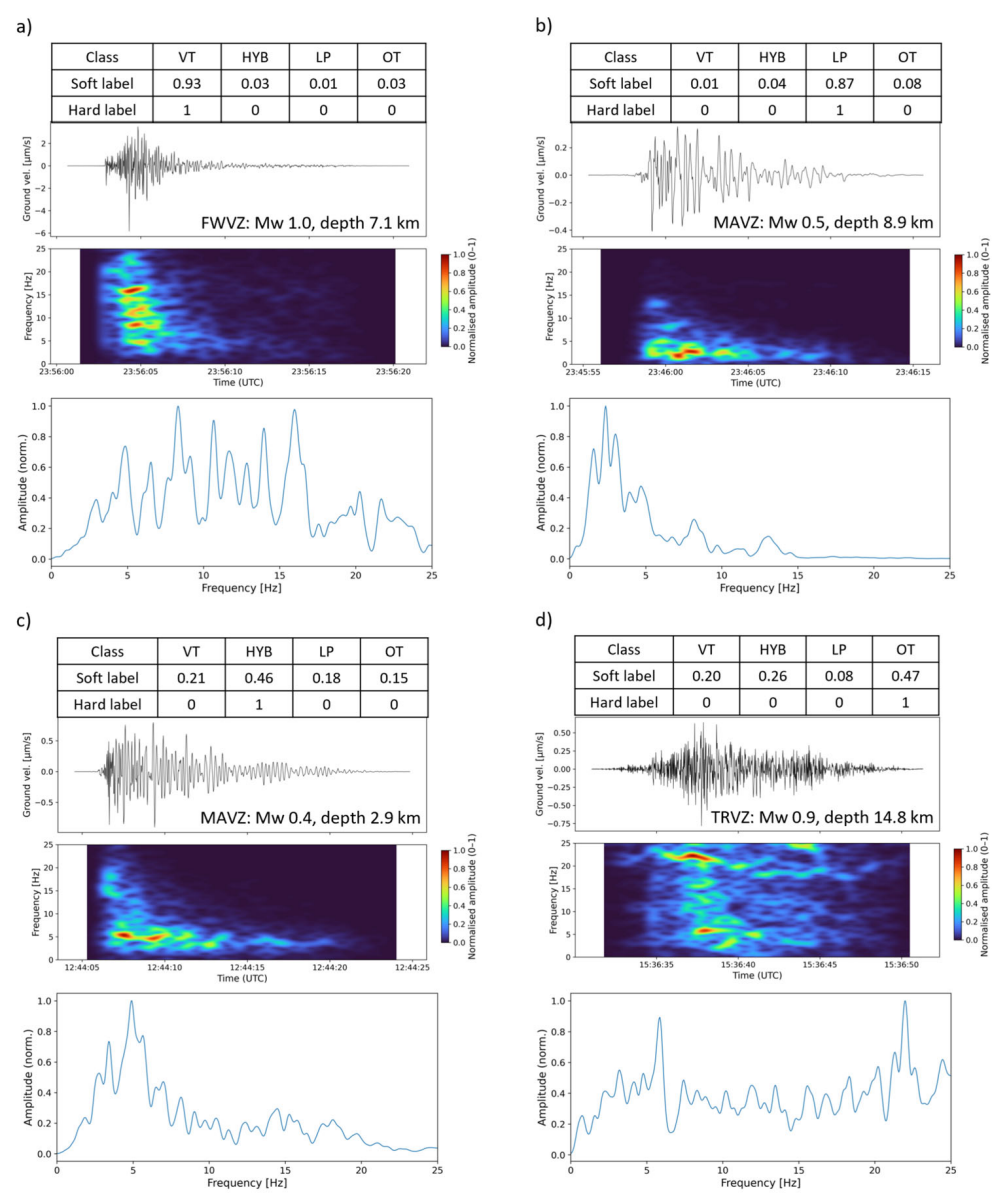
Data streams of events sampled from batches 2, 3, and 4 showing the probabilistic soft labels and equivalent hard labels for each event derived from crowdsourced expert judgement. Event **a** (batch 4 question 7) epicentre was 2 km from FWVZ station (NW of Ruapehu summit) and has a 93% likelihood of being a VT event. Event **b** (batch 2 question 3) epicentre was 4.3 km from MAVZ station (Ruapehu summit, north) and has a 87% likelihood of being an LP. Event **c** (batch 3 question 7) epicentre was 0.4 km from MAVZ station (Ruapehu summit, north) and has a 46% likelihood of being a HYB event. Event **d** (batch 4 question 6) epicentre was 2.4 km from TRVZ station (SW of Ruapehu summit) and has a 47% likelihood of being a signal that does not fit to the standard classification

In Fig. 7, we present four events sampled from the questionnaire batches to illustrate the representation of soft labelling for the volcano-seismic data. The examples were selected based on the strength of the consensus toward one of the four classifications, i.e. whether it was generally considered a VT, HYB, LP, or OT event. There is generally a stronger consensus within each batch on the classification of VT and LP events, which can also be seen in Fig. 5.

**Fig. 8:**
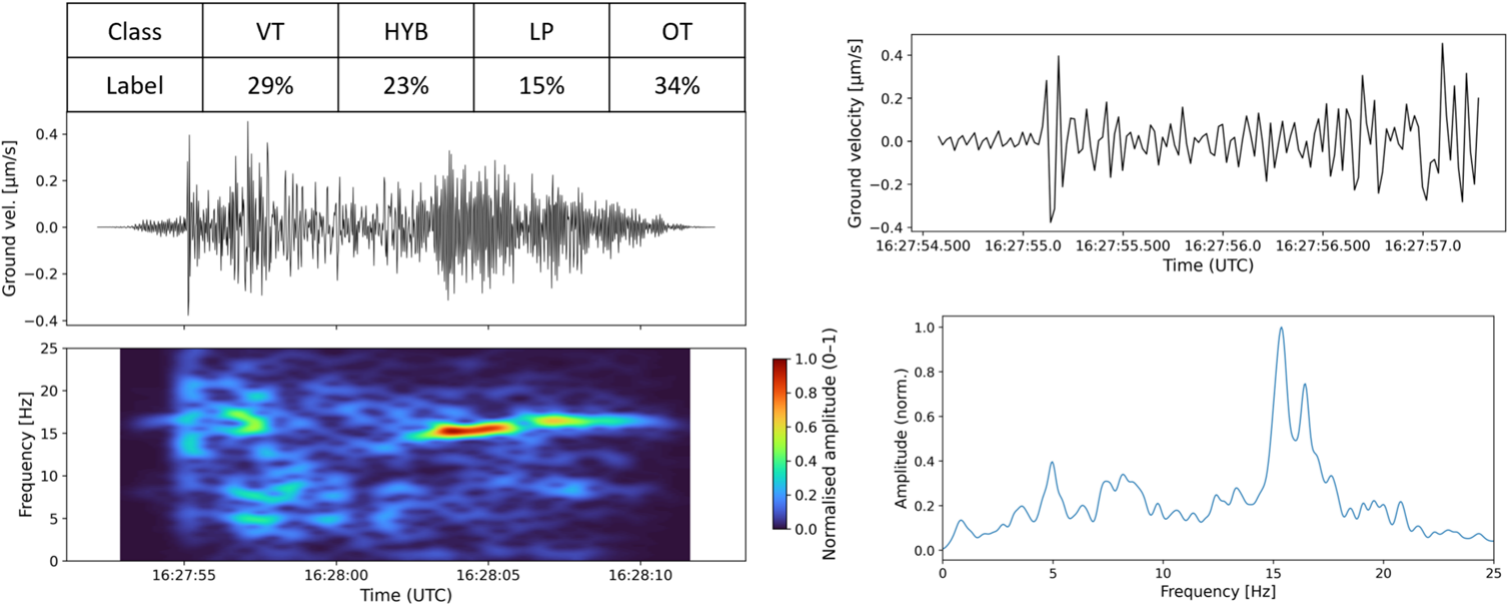
Question 2 from batch 5 shown in trace, onset zoom, spectrogram, and spectrum form, with constructed probabilistic soft labels. The Mw 0.5 event epicentre was approximately 4.2 km from FWVZ station at 16:27:52UTC on 08/09/2007 at a depth of 10.4 km

Some events showed genuine ambiguity among experts in each batch (e.g. Fig. 8). The survey found that there were approximately 21% of events in the questionnaire where a single label does not produce a probability of >50%. Question 2 in batch 5 (Fig. 8) had comments included by the annotators that also inferred potential ambiguity in the signal. Two experts believed the trace could be showing two separate events, perhaps a VT earthquake triggering another type, with another commenting that this could be a volcano-seismic signal with transient noise. Another annotator described this event as tremor.

## Discussion

The classification of volcano-seismic signals is a single component within a broader field of probabilistic hazard and risk assessment, which includes detailed hazard scenarios (Newhall and Pallister 2015; Bebbington 2014) and eruption forecasting models, such as event trees (Newhall and Hoblitt 2002; Neri et al. 2008; Newhall and Pallister 2015) or Bayesian networks (Christophersen et al. 2022) at active volcanoes. In fact, structured elicitation that combines expert estimates to address uncertainties and biases in expert judgement is a relatively mature process and plays an important role in many decision-making practices in volcanology (Aspinall et al. 2003; Aspinall 2006, 2010; Tadini et al. 2022).

It could be that volcano-seismic events exist along a frequency spectrum ranging from VT to LP classifications, making the categorisation of these signals into discrete bins a fundamental problem. Naturally, this has led to explorations for alternative methods such as the development of statistical descriptions of signals using the ratio of high and low-frequency energy (Buurman and West 2010) or the clustering of latent mathematical features of the signal (e.g. Duque et al. 2020; Cui et al. 2021). We have shown that these boundary events that may have an ambiguous label can be captured using the soft labelling approach that represents the expert consensus and therefore label confidence. For volcano-seismic events in the study, there is no established ground truth, which prevents us from measuring the performance of each expert and weight accordingly (e.g. Aspinall et al. 2003). Indeed, assessing individual expert accuracy is essentially impossible to measure for this task and may even be irrelevant (e.g. Cholleti et al. 2009). The lack of a gold standard label has major implications for machine learning classifiers, be it due to inherent noise commonly found in agreement statistics or derived from disagreement in the fundamental classification terms for these signals. What has been shown is how well a panel of experts agree on a single task, and the variability of these judgements is what has been termed noise (Kahneman et al. 2021). Contextual information for each volcano-seismic event was limited to the magnitude, depth, and distance to the volcano summit and seismic station. Some experts expressed the need for more context regarding the signal they were observing. On the one hand, understanding the context of the signal is extremely beneficial for interpreting what kind of source mechanisms may drive earthquakes in the region. However, we were wary that depending on context may unconsciously bias the judgement of the expert towards prior experiences. Instead, it was integral to the study that each individual base their judgement solely on the data streams provided. We used the ICC to assess whether experts form a consistent agreement by consensus, and the results indicated that within every volcano-seismic category, there is considerable uncertainty among experts. However, we observe good to excellent reliability when averaging across multiple experts, particularly for VT and LP events, and even moderate reliability for HYB events. Machine learning serves as a tool to help automate some of the processes that formulate expert judgement. However, SML models depend on the expert judgement to construct training data sets for the model to learn from. While single-expert classifications provide a useful forecasting tool, our results highlight the risk of relying solely on the interpretation of one expert when training machine learning models.

Volcano-seismic classifications were originally established by a select group of experts who made observations and interpretations based on a limited number of volcanoes (e.g. Minakami et al. 1951; Latter 1981; Chouet 1986; Lahr et al. 1994). The source-mechanism models relating to these signals have profound implications for interpretations of processes occurring in the volcanic system (e.g. Sparks 2003; Chouet and Matoza 2013; Matoza and Roman 2022). However, they are applied at numerous volcanoes worldwide without a formal calculation of uncertainty (Sparks and Aspinall 2004). VT swarms have been one of the most widely used precursory signals for eruptions, but with occasional contrasting precursory relevance (Roman and Power 2011). The cumulative magnitude of VT swarms has even been proposed as a proxy for magma intrusion volume estimates (White and McCausland 2016; Meyer et al. 2021; Danré et al. 2022). HYB swarms have been associated with lava dome growth and even a precursory signal for dome collapse at Soufriere Hills Volcano (Miller et al. 1998; Ottemöller 2008). The source mechanics relating to LP earthquakes are often linked to gas and fluid movement (Chouet 1996; Clarke et al. 2021), which perhaps has the most significant implications for volcanic unrest, but can also be interpreted as slow faulting (Bean et al. 2014). It was not the objective of this study to deny the validity of these precursors. However, the unreliable agreement between experts in volcano-seismology emphasises the importance of understanding the uncertainty in classifying these signals. This is particularly significant, as both volcano monitoring and broader research into volcanic processes rely on these event classifications to yield insights into the dynamics of the volcanic system (Cortés et al. 2021).

### Expert agreement

The primary objective of this research was to evaluate the reliability of expert judgement in classifying a single transient volcano-seismic signal, where the expert must rely solely on the data and limited contextual background. The results show that under these conditions, which are comparable to a volcano showing signs of unrest after a period of repose, where only a single station is operational, expert agreement can vary significantly. Identical measurements between raters would yield an ICC(2,k) score of 1. The highest single-point agreement estimate was 0.94 with relatively narrow 95% confidence bands (0.72–0.96) between experts in batch 1 for labelling VTs only (Table 2). This is an excellent agreement (Koo and Li 2016); however, it falls significantly to 0.54 (moderate agreement) when using the single-rater model. In general, the single-rater model (ICC (2,1)) is found to produce a lower absolute agreement than the mean (ICC (2,k)) (Koo and Li 2016), i.e. in statistics, the mean score of a group of raters tends to outperform individual judgement (e.g. wisdom of the crowd) (Rauhut and Lorenz 2011; Nascimento et al. 2022; Schmarje et al. 2022). Similar studies have found that collective decision-making does not outperform the aggregated average judgement of individuals (Hamada et al. 2020). This is also in agreement with Wright and Augenstein (2025), who found aggregating existing soft labelling methods both improved accuracy and tightened calibration, even across different domains.

The ICC values are driven by between-event variability as well as the variability within each rater’s measurements (Koo and Li 2016). We believe that 10 individual events with three potential categories (and one category attributed to non-volcanic signals) were sufficient to provide true event variability within each batch. We therefore infer that the low single-rater ICC(2,1) scores are due to genuine variance in expert judgement. In such cases, it might be recommended to use the mean because individual ratings are too unreliable (Shrout and Fleiss 1979). There is generally good agreement for VT and LP events when averaging at least 4–5 raters, which are fundamental labels for volcano-seismic machine learning classifiers (Canário et al. 2020; Bueno et al. 2020; Manley et al. 2022), with HYB labels either not used or combined with other mixed seismic signatures (Bueno et al. 2020). The significant variability observed among experts suggests that there may be no definitive ground truth label for volcano-seismic events. In fact, some expert feedback expressed that the current classification scheme is impractical for volcano monitoring, and it has been shown that the physical processes driving these signals may not fit the standard volcano-seismic classification labels (Matoza and Roman 2022). Given that they remain critical for monitoring and forecasting, reducing noise in expert judgement requires the revision of the current classification scheme towards developing a more robust gold standard, or by introducing frequent noise audits utilising ICCs or an alternative agreement metric (Nascimento et al. 2022).

#### Does experience matter?

We segmented each batch by experience to investigate potential correlations between years of experience and agreement. Each participant was asked to select from the following experience buckets: postgraduate student, 1–5 years, 5–10 years, 10–20 years, >20 years. We found that there was no clear positive correlation, but there were perhaps some nuances worth commenting on. We do observe a slight increase in reliability for single-rater scores for HYB events with experience, but not enough to deem reliable volcano-seismic classification. Similarly for VT and LP, we never see a consensus that merits a good single-rater score. This means that although it is recommended that to reliably classify volcano-seismic events one should consider using at least four experts, it matters less whether the pool of experts contains senior or early-career researchers. Therefore, we can say that the variability in expert judgement is not driven by inexperience but is underpinned by genuine ambiguity in the data with regards to the given discrete labels.

At the end of the questionnaire, we asked each participant to rate how useful each data stream was to assist the expert in their judgement. We found that there was a general consensus in the ranking of the trace, spectrogram, and spectrum for experts with >1 year experience, where the most useful data stream was the spectrogram, followed by the trace, and the least useful data stream was the spectrum. However, we found that with increasing experience, the margin between the spectrogram and the trace decreases, and for the most experienced experts, the trace becomes the most useful data source on average. For the most experienced experts (>20 years), the standard deviation for the scores of the three data sources was lowest (0.84), implying that senior experts were more likely to make judgements equally based on the three data streams, whereas the early career experts were generally more dependent on the spectrogram. This could be due to computational constraints pre–2000, where spectrograms were relatively hard to create and visualise; therefore, experienced researchers may be more used to using the waveform traces and spectra to do volcano-seismic classification.

### Applications for machine learning

For SML, input data is transformed into vectors before being processed, and the vectorisation method selected for training depends on the nature of the input data and the model being used. It is difficult to comment on the labelling method used in volcano-seismic classification literature because it is rarely explicitly detailed. However, the use of experts to apply a discrete classification to a signal is commonly mentioned (Malfante et al. 2018; Falcin et al. 2021; Manley et al. 2022), which is likely a form of one-hot encoding or hard labelling (Poslavskaya and Korolev 2023). Machine learning models trained on hard labels learn to produce highly confident scores for each prediction, which breaks down when there is no clear boundary between classes (Vega et al. 2021). Therefore, noisy or even mislabelled data can lead to adverse estimates of model accuracies. We observe this in volcano-seismic data, and it is demonstrated via the inter-rater reliability results. We have shown that reliability in labelled data sets should not be taken for granted. Instead, volcano-seismic classification labels should also be treated probabilistically to incorporate measured uncertainty directly into the model. This is analogous to methods in medical imaging that combine the opinions of multiple experts to train more reliable models (Silva and Oliveira 2021; Vega et al. 2021). The exploration of Bayesian neural networks (BNNs) to capture model uncertainty in volcano-seismic classification (Bueno et al. 2020) shows a shift from deterministic to probabilistic modelling (Sparks and Aspinall 2004). Our results show how a simple MAP framework can incorporate expert agreement to create soft labelled data sets, which could enhance current models towards a fully probabilistic framework.

Soft labels can be incorporated into ML pipelines for a variety of models that have already shown promise for volcano-seismic classification, such as convolutional neural networks (e.g. Manley et al. 2022) and BNNs (e.g. Bueno et al. 2020). This can be achieved by changing the target format from one-hot to probability distribution vectors and ensuring that the loss function has soft label compatibility (e.g. Ge et al. 2022; Nousi and Tefas 2024). The adoption of probabilistic soft labelling elicited from even a small pool of experts (e.g. Collins et al. 2022), and shown in Fig. 4, could present an opportunity to greatly improve model accuracy and calibration (Vega et al. 2021). Furthermore, models trained on soft labels have been found to generalise better to unseen data (Vyas et al. 2021), meaning that classifiers trained at one volcano could be transferable to other volcanoes. Lee et al. (2022) showed how cross-domain generated soft labelling achieved up to +6.9 pp on a standard *leave-one-domain-out* benchmark compared to hard labelling. This means that combining datasets from a variety of volcanoes could help reduce the burden of data volume for model training whilst potentially improving model generalisability.

We recognise that time constraints limit the feasibility of large-scale, multi-analyst labelling of volcano-seismic data. We therefore propose periodic audits led by a small group of experts to produce a subset of soft labels. The resulting uncertainties quantified in the audit can be applied in practice as prior probabilities or calibration targets downstream in ML models. Further data augmentation can be achieved by collaborating with observatories and research groups to construct cross-domain (multi-volcano) datasets, which could reduce the need for future large-scale labelling efforts.

### Limitations

With this being the first attempt to crowdsource expert judgement on volcano-seismic classification, it is anticipated that there may be areas where the methodology can be improved. Moreover, although methods for assessing expert agreement, such as the ICC, have been evaluated and implemented in disciplines such as psychology and medical sciences, there are no universally established procedures in this context. While the application of soft labels in machine learning classifiers appears promising, the methodologies for creating soft labels remain an area of development. Part of the survey enabled experts to provide their opinions on the design of the methodology, and the general feedback was positive. We have noted how the path effect when seismic energy travels from source to station could result in signal attenuation and aimed to mitigate this by selecting the closest station to the event. However, experts expressed uncertainty on whether the seismic attenuation had affected some of the events illustrated in the questionnaire. We recognise the importance of applying methodologies, such as stacked spectra from three or more stations, to separate the *true* signal from source and path effects. However, the option to utilise multiple stations at a volcano is not always available. Furthermore, the ability to adapt time windows would help experts distinguish between transient noise and volcano-seismic signals, which may be present in the data (Fig. 8). The integration of soft labelling functionality into software such as SWARM could allow for the extension of labels for continuous signals; this would also allow for the user to utilise multiple stations.

There is a possibility that the questionnaire could be interpreted differently depending on the expert. In particular, the sliding scale to select a probability label from $-1$ to $+1$ for all four classes could be difficult to understand, and a simpler framework may reduce inter-rater noise whilst still producing reliable soft labels. Finally, although we provided evidence that using multiple annotators to construct probabilistic soft labels would be beneficial, we did not have enough data to perform an actual machine learning benchmark.

## Summary

The aim of this study was to assess the level of agreement between experts when undertaking an everyday but non-trivial task. Classifying volcano-seismic events is essential both for operational monitoring and for advancing fundamental volcanic research; thus, ensuring close agreement among experts is vital to achieve accurate risk assessments. We presented a method for soft labelling and inter-rater uncertainty that is both novel for volcano-seismology and supported by cross-disciplinary evidence, which could lead to machine learning models that produce reliable accuracy claims and are able to generalise well to other volcanoes. The observation of disagreement among experts from different volcanic settings is of great significance for current monitoring practices and emphasises the requirement for multiple experts to produce reliable judgements. Future work may benefit from quantifying the agreement using data from different volcanic settings. We have shown how volcano-seismic classification can be achieved using crowdsourcing through the use of an online questionnaire. We have also shown how aggregating the judgements of experts from different volcanic background can result in a somewhat reliable consensus, so one does not have to depend solely upon local expert knowledge to perform these tasks. Studies showing the benefits of cross-domain training for model generalisation (e.g. Lee et al. 2022), along with methods for reducing training data volumes while improving model accuracy (e.g. Manley et al. 2022), make the utilisation of multiple experts and the calculation of their uncertainty feasible for volcano-seismic classification. For machine learning, the accuracy of the model output is restricted to the reliability of the labelled data. If the training data is of high quality and incorporates uncertainty, then the model should produce reliable outputs, which may generalise well to other volcanoes. This is certainly of scientific interest and merits future work.

## Supplementary Information

Below is the link to the electronic supplementary material.

Supplementary file 1 (pdf 636 KB)

## Data Availability

A sample of the full earthquake catalogue from Illsley-Kemp and Mestel (2025) was used in this study. The full catalogue is freely available at https://doi.org/10.5281/zenodo.13138604. The GeoNet seismic data is freely available through GeoNet.

## Code Availability

Available on request.
