# Supplementary material for: Capturing expert uncertainty: ICC-informed soft labelling for volcano-seismicity
Source: Bull Volcanol. 2025 Sep 16;87(10):84. doi: 10.1007/s00445-025-01875-4 (PMC12441074; doi:10.1007/s00445-025-01875-4)
Supplement: Supplementary file 1 — (pdf 636 KB) [file 445_2025_1875_MOESM1_ESM.pdf]

# 1 Supplementary Material

## 1.1 Questionnaire

For the event selection, we selected a stratified sample of 40 *high frequency* events (five per questionnaire) with FI values $> 0.4$, because we saw a dominant peak for FI 0.5–0.75. We selected 16 events (two per questionnaire) with a FI $< 0$ showing spectral energies in the *low frequency* range. we chose 16 events because although the catalogue was imbalanced towards VT events, we wanted at least two events per questionnaire to have dominant energy for frequencies <5 Hz. The remaining 24 events were sampled from the events (three per questionnaire) that fit FI 0–0.4 so could contain low and high frequencies and possibly hybrid events. This sampling was representative of the FI distribution and ensures exposure to the full range of frequency signals within each questionnaire batch (Figure 1). This created a subset of 80 individual events to be used in the questionnaire (10 events per questionnaire). We believed that 80 events would be representative of the general seismicity of the area of interest.

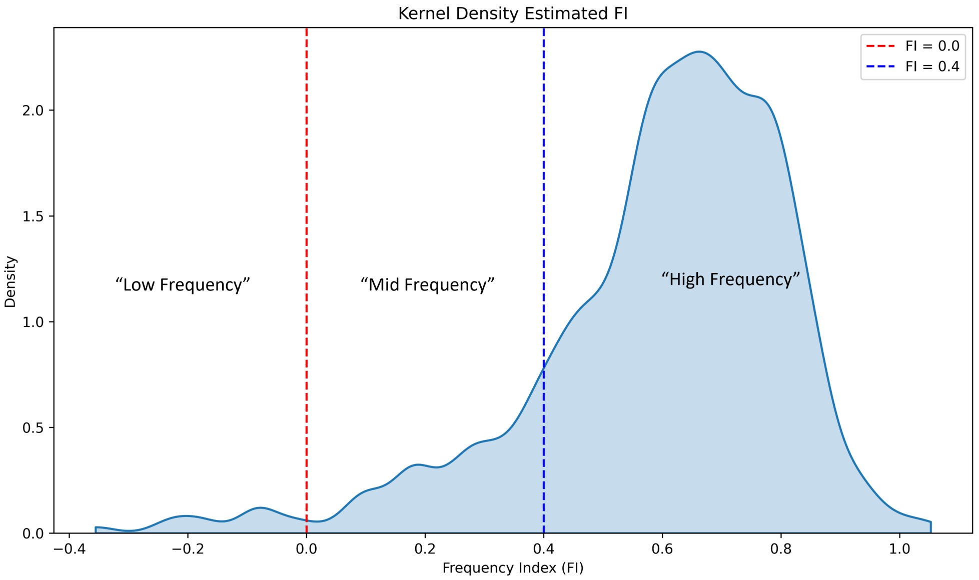


**Fig. 1** Kernel Density Estimated Frequency Index using the closest station to earthquake hypocentre. FI calculation bands: Lower = 1–5 Hz, Upper = 5–20 Hz.

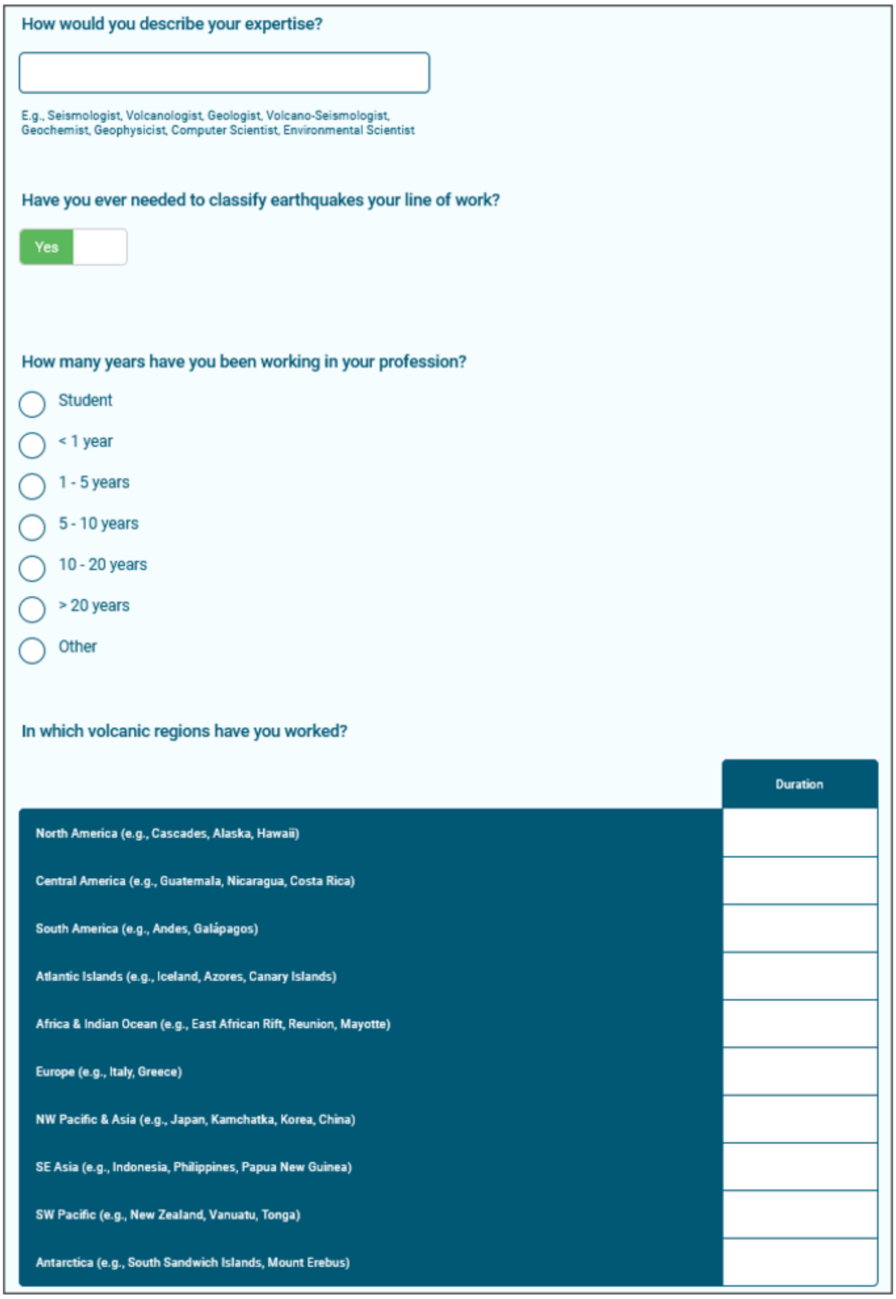

How would you describe your expertise?

E.g., Seismologist, Volcanologist, Geologist, Volcano-Seismologist, Geochemist, Geophysicist, Computer Scientist, Environmental Scientist

Have you ever needed to classify earthquakes your line of work?

Yes

How many years have you been working in your profession?

- Student
- < 1 year
- 1 - 5 years
- 5 - 10 years
- 10 - 20 years
- > 20 years
- Other

In which volcanic regions have you worked?

| | Duration |
|---|---|
| North America (e.g., Cascades, Alaska, Hawaii) | |
| Central America (e.g., Guatemala, Nicaragua, Costa Rica) | |
| South America (e.g., Andes, Galápagos) | |
| Atlantic Islands (e.g., Iceland, Azores, Canary Islands) | |
| Africa & Indian Ocean (e.g., East African Rift, Reunion, Mayotte) | |
| Europe (e.g., Italy, Greece) | |
| NW Pacific & Asia (e.g., Japan, Kamchatka, Korea, China) | |
| SE Asia (e.g., Indonesia, Philippines, Papua New Guinea) | |
| SW Pacific (e.g., New Zealand, Vanuatu, Tonga) | |
| Antarctica (e.g., South Sandwich Islands, Mount Erebus) | |

**Fig. 2** This figure shows the questions asked about the background experience for each expert taken from the online questionnaire

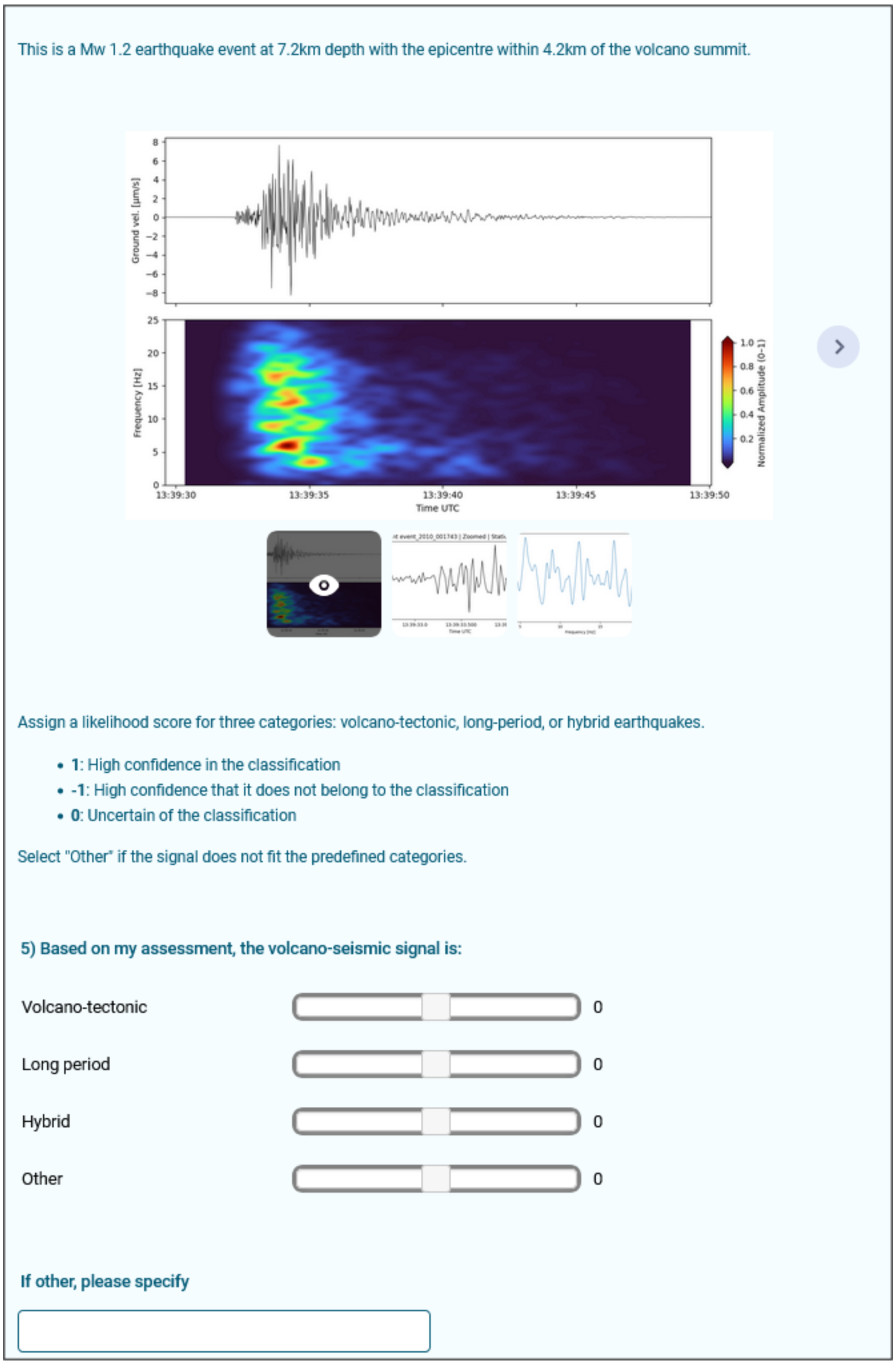


**Fig. 3** This figure shows a sample question from the online questionnaire. The expert is given a summary of the earthquake event and an image slider to rotate through data formats. The expert then judges the likelihood of that event belonging to each of the four categories using the sliders for each classification.

## 1.2 ICC calculation

For the ICC full calculation we have $n$ subjects (events) and $k$ raters. The score given by rater $j$ on subject $i$ is denoted $x_{ij}$. In our example $n = 10$, $k = 12$.

The means for raters, subjects and total mean:

$$\bar{x}_{\bullet\bullet} = \frac{1}{n\,k}\sum_{i=1}^{n}\sum_{j=1}^{k} x_{ij} \tag{1}$$

$$\bar{x}_{i\bullet} = \frac{1}{k}\sum_{j=1}^{k} x_{ij} \quad (i = 1, \ldots, n) \tag{2}$$

$$\bar{x}_{\bullet j} = \frac{1}{n}\sum_{i=1}^{n} x_{ij} \quad (j = 1, \ldots, k) \tag{3}$$

The sum of the squares for events, raters and the residual error:

$$\mathrm{SS}_{\text{subjects}} = k\sum_{i=1}^{n}\big(\bar{x}_{i\bullet} - \bar{x}_{\bullet\bullet}\big)^2 \tag{4}$$

$$\mathrm{SS}_{\text{raters}} = n\sum_{j=1}^{k}\big(\bar{x}_{\bullet j} - \bar{x}_{\bullet\bullet}\big)^2 \tag{5}$$

$$\mathrm{SS}_{\text{error}} = \sum_{i=1}^{n}\sum_{j=1}^{k}\Big[x_{ij} - \bar{x}_{i\bullet} - \bar{x}_{\bullet j} + \bar{x}_{\bullet\bullet}\Big]^2 \tag{6}$$

Finally, the mean squares is calculated from the degrees of freedom for ANOVA models: Degrees of freedom:

$$\mathrm{df}_{\text{subjects}} = n - 1, \quad \mathrm{df}_{\text{raters}} = k - 1, \quad \mathrm{df}_{\text{error}} = (n-1)(k-1).$$

$$\mathrm{MS}_{\text{subjects}} = \frac{\mathrm{SS}_{\text{subjects}}}{n-1}, \tag{7}$$

$$\mathrm{MS}_{\text{raters}} = \frac{\mathrm{SS}_{\text{raters}}}{k-1}, \tag{8}$$

$$\mathrm{MS}_{\text{error}} = \frac{\mathrm{SS}_{\text{error}}}{(n-1)(k-1)}. \tag{9}$$

For the *average* of $k$ raters in a two-way random-effects, absolute-agreement model:

$$\mathrm{ICC}(2,k) = \frac{\mathrm{MS}_{\text{subjects}} - \mathrm{MS}_{\text{error}}}{\mathrm{MS}_{\text{subjects}} + \dfrac{\mathrm{MS}_{\text{raters}} - \mathrm{MS}_{\text{error}}}{n}}. \tag{10}$$

For $n = 10$, $k = 12$, and the computed mean squares values for VT (batch 1):

$$\mathrm{MS}_{\text{subjects}} = 3.1, \quad \mathrm{MS}_{\text{raters}} = 0.37, \quad \mathrm{MS}_{\text{error}} = 0.18, \quad n = 10.$$

Then

$$\mathrm{ICC}(2,12) = \frac{3.1 - 0.18}{3.1 + \frac{0.37-0.18}{10}} \approx 0.94$$

## 1.3 Soft label dataset

**Table 1**: Per-event soft labels (probabilities) for all batches ($T$=0.3).

| Batch | Event | VT | LP | HYB | OT |
|---|---|---|---|---|---|
| b1 | 1 | 0.83 | 0.01 | 0.08 | 0.08 |
| b1 | 2 | 0.76 | 0.04 | 0.08 | 0.11 |
| b1 | 3 | 0.45 | 0.18 | 0.18 | 0.18 |
| b1 | 4 | 0.02 | 0.82 | 0.06 | 0.11 |
| b1 | 5 | 0.02 | 0.82 | 0.06 | 0.10 |
| b1 | 6 | 0.86 | 0.02 | 0.04 | 0.08 |
| b1 | 7 | 0.12 | 0.28 | 0.41 | 0.19 |
| b1 | 8 | 0.84 | 0.02 | 0.05 | 0.09 |
| b1 | 9 | 0.68 | 0.04 | 0.13 | 0.14 |
| b1 | 10 | 0.31 | 0.15 | 0.35 | 0.18 |
| b2 | 1 | 0.83 | 0.01 | 0.07 | 0.09 |
| b2 | 2 | 0.77 | 0.02 | 0.08 | 0.13 |
| b2 | 3 | 0.01 | 0.87 | 0.04 | 0.08 |
| b2 | 4 | 0.64 | 0.04 | 0.17 | 0.15 |
| b2 | 5 | 0.16 | 0.22 | 0.44 | 0.18 |
| b2 | 6 | 0.87 | 0.01 | 0.04 | 0.08 |
| b2 | 7 | 0.67 | 0.03 | 0.17 | 0.13 |
| b2 | 8 | 0.73 | 0.03 | 0.12 | 0.13 |
| b2 | 9 | 0.03 | 0.61 | 0.18 | 0.17 |
| b2 | 10 | 0.15 | 0.47 | 0.21 | 0.17 |
| b3 | 1 | 0.88 | 0.02 | 0.03 | 0.06 |
| b3 | 2 | 0.06 | 0.54 | 0.29 | 0.11 |
| b3 | 3 | 0.45 | 0.09 | 0.29 | 0.17 |
| b3 | 4 | 0.05 | 0.72 | 0.11 | 0.13 |
| b3 | 5 | 0.62 | 0.06 | 0.19 | 0.13 |
| b3 | 6 | 0.10 | 0.54 | 0.23 | 0.13 |

*Continued on next page*

| Batch | Event | VT | LP | HYB | OT |
|---|---|---|---|---|---|
| b3 | 7 | 0.21 | 0.18 | 0.46 | 0.15 |
| b3 | 8 | 0.57 | 0.09 | 0.21 | 0.13 |
| b3 | 9 | 0.66 | 0.06 | 0.17 | 0.10 |
| b3 | 10 | 0.75 | 0.05 | 0.10 | 0.10 |
| b4 | 1 | 0.63 | 0.02 | 0.27 | 0.09 |
| b4 | 2 | 0.63 | 0.02 | 0.25 | 0.10 |
| b4 | 3 | 0.87 | 0.01 | 0.06 | 0.06 |
| b4 | 4 | 0.01 | 0.90 | 0.05 | 0.04 |
| b4 | 5 | 0.01 | 0.86 | 0.04 | 0.09 |
| b4 | 6 | 0.20 | 0.08 | 0.26 | 0.47 |
| b4 | 7 | 0.93 | 0.01 | 0.03 | 0.03 |
| b4 | 8 | 0.66 | 0.05 | 0.16 | 0.13 |
| b4 | 9 | 0.82 | 0.01 | 0.08 | 0.08 |
| b4 | 10 | 0.16 | 0.42 | 0.30 | 0.13 |
| b5 | 1 | 0.80 | 0.02 | 0.10 | 0.08 |
| b5 | 2 | 0.29 | 0.15 | 0.23 | 0.34 |
| b5 | 3 | 0.01 | 0.89 | 0.04 | 0.07 |
| b5 | 4 | 0.56 | 0.06 | 0.26 | 0.12 |
| b5 | 5 | 0.76 | 0.02 | 0.11 | 0.11 |
| b5 | 6 | 0.49 | 0.09 | 0.22 | 0.20 |
| b5 | 7 | 0.07 | 0.59 | 0.23 | 0.11 |
| b5 | 8 | 0.02 | 0.85 | 0.07 | 0.07 |
| b5 | 9 | 0.88 | 0.01 | 0.04 | 0.07 |
| b5 | 10 | 0.56 | 0.04 | 0.26 | 0.14 |
| b6 | 1 | 0.79 | 0.04 | 0.09 | 0.08 |
| b6 | 2 | 0.83 | 0.03 | 0.08 | 0.07 |
| b6 | 3 | 0.87 | 0.02 | 0.06 | 0.06 |
| b6 | 4 | 0.66 | 0.06 | 0.16 | 0.12 |
| b6 | 5 | 0.34 | 0.30 | 0.19 | 0.16 |
| b6 | 6 | 0.08 | 0.51 | 0.23 | 0.18 |
| b6 | 7 | 0.05 | 0.72 | 0.11 | 0.12 |
| b6 | 8 | 0.54 | 0.14 | 0.19 | 0.14 |
| b6 | 9 | 0.12 | 0.48 | 0.21 | 0.19 |
| b6 | 10 | 0.06 | 0.72 | 0.10 | 0.13 |
| b7 | 1 | 0.88 | 0.02 | 0.03 | 0.07 |
| b7 | 2 | 0.43 | 0.05 | 0.35 | 0.16 |
| b7 | 3 | 0.71 | 0.07 | 0.10 | 0.13 |
| b7 | 4 | 0.06 | 0.64 | 0.17 | 0.13 |
| b7 | 5 | 0.04 | 0.81 | 0.06 | 0.10 |
| b7 | 6 | 0.05 | 0.79 | 0.06 | 0.11 |
| b7 | 7 | 0.63 | 0.11 | 0.13 | 0.13 |
| b7 | 8 | 0.49 | 0.06 | 0.32 | 0.14 |
| b7 | 9 | 0.79 | 0.03 | 0.09 | 0.09 |
| b7 | 10 | 0.84 | 0.03 | 0.05 | 0.08 |
| b8 | 1 | 0.83 | 0.04 | 0.07 | 0.07 |
| b8 | 2 | 0.79 | 0.05 | 0.08 | 0.09 |
| b8 | 3 | 0.63 | 0.10 | 0.13 | 0.13 |

*Continued on next page*

| Batch | Event | VT | LP | HYB | OT |
|---|---|---|---|---|---|
| b8 | 4 | 0.28 | 0.24 | 0.29 | 0.19 |
| b8 | 5 | 0.16 | 0.52 | 0.17 | 0.16 |
| b8 | 6 | 0.77 | 0.05 | 0.09 | 0.10 |
| b8 | 7 | 0.25 | 0.38 | 0.19 | 0.18 |
| b8 | 8 | 0.84 | 0.03 | 0.06 | 0.07 |
| b8 | 9 | 0.69 | 0.06 | 0.13 | 0.12 |
| b8 | 10 | 0.05 | 0.78 | 0.08 | 0.09 |
